# Integrative bioinformatics analysis identifies gut microbiota-derived metabolite-associated immune candidate targets in high-risk primary biliary cholangitis

**DOI:** 10.1371/journal.pone.0356008

**Published:** 2026-08-13

**Authors:** Jing Feng, Zhiyun Liu, Huanna Sun

**Affiliations:** 1 First Clinical Medical College, Shandong University of Traditional Chinese Medicine, Jinan, China; 2 Affiliated Hospital of Shandong University of Traditional Chinese Medicine, Jinan, China; Charite Universitatsmedizin Berlin, GERMANY

## Abstract

**Objective:**

This study aimed to identify and prioritize gut microbiota-derived metabolite-associated host candidate targets in high-risk primary biliary cholangitis (PBC) through integrative multi-omics analysis and external validation.

**Methods:**

Differentially expressed genes between high-risk and low-risk PBC liver samples were identified using GSE79850. Gut microbiota-derived metabolite-host gene associations and PBC-related targets were obtained from gutMGene and the Open Targets Platform, respectively. Candidate genes were identified through integrative intersection analysis and ranked using an exploratory priority score. Human Liver Cell Atlas-based reference localization, immune signature analysis, and external validation using GSE119600 were performed to further characterize the candidate genes.

**Results:**

A total of 163 differentially expressed genes were identified in GSE79850. Integrative analysis with gutMGene and the Open Targets Platform identified six core candidate genes: CCL2, CDKN1A, BCL2, CD44, FOS, and MAPK3. CCL2 showed the highest priority score, was associated with multiple gut microbiota-related metabolites, including acetate, butyrate, propionate, and succinate, and was significantly upregulated in high-risk PBC liver samples. Human Liver Cell Atlas-based reference localization showed that CCL2 was mainly distributed in immune-related cell populations, including macrophages and monocyte-derived cells. High-risk PBC samples exhibited increased chemokine signaling signature scores, and CCL2 expression was positively correlated with this signature. In the GSE119600 cohort, CD44, FOS, and CDKN1A were significantly upregulated in PBC samples compared with controls.

**Conclusion:**

This integrative analysis identified six candidate host targets associated with high-risk PBC. CCL2 showed the most convergent evidence within the discovery framework and may represent a chemokine-related hepatic microenvironment-associated candidate target. In contrast, CD44, FOS, and CDKN1A showed supportive expression patterns in the GSE119600 blood cohort. These findings represent exploratory candidate prioritization, and further validation in independent liver tissue cohorts and experimental studies is warranted.

## Introduction

### Primary biliary cholangitis and high-risk disease

Primary biliary cholangitis (PBC) is a chronic autoimmune cholestatic liver disease characterized by progressive immune-mediated injury of the small intrahepatic bile ducts. Persistent cholestasis and chronic intrahepatic inflammation may lead to liver fibrosis, cirrhosis, and eventually end-stage liver disease in a subset of patients. Ursodeoxycholic acid (UDCA) remains the first-line therapy for PBC and improves long-term outcomes in many patients. However, a considerable proportion of patients show inadequate biochemical response or remain in a high-risk state, which is associated with a higher risk of disease progression. Therefore, early identification of molecular features and risk-associated candidate targets remains important for subsequent validation studies [1,2].

Risk stratification in PBC has traditionally relied largely on serum biochemical indicators, while molecular features in liver tissue may provide additional information. Using liver transcriptomic data obtained at diagnosis, Hardie et al. reported that high-risk and low-risk PBC patients already showed distinct molecular expression patterns at an early disease stage, suggesting that liver transcriptomics may help identify patients at increased risk of subsequent disease progression [3]. However, differential expression analysis alone mainly answers which genes are altered, but does not clarify whether these genes are linked to gut microbiota-derived metabolites, disease-target evidence, or specific liver cell types. This limits the extension of differential gene findings toward mechanistic hypotheses and downstream experimental validation.

### The gut-liver axis and gut microbiota-derived metabolites

The gut-liver axis provides an important entry point for understanding the immune-inflammatory environment of PBC. Gut microbiota and their metabolites communicate continuously with the liver through the portal venous system and may influence bile acid metabolism, intrahepatic immune regulation, inflammatory responses, and tissue repair. Previous studies have shown that patients with PBC have altered gut microbial profiles, and some of these microbial features may be partially restored after UDCA treatment. Treatment-naive PBC patients also show microbial profiles distinct from those of healthy individuals [4,5].

Beyond microbial composition, gut microbiota-derived metabolites may also be involved in the disease state of PBC. Short-chain fatty acid profiles and fecal microbiota features have been reported to be associated with fibrosis severity in PBC, suggesting that metabolite-level alterations may be related to disease severity [6]. Recent evidence further indicates that gut microbiota and metabolite signatures are associated with poor biochemical response after UDCA treatment in PBC [7]. These findings suggest that gut microbiota-derived metabolites may contribute to hepatic immune and inflammatory regulation through the gut-liver axis. At present, however, such evidence mainly supports associations and should not be interpreted as direct proof of causality. Therefore, the relationship between microbial metabolites and high-risk PBC should be interpreted with appropriate caution.

### Knowledge gap

Overall, existing studies have advanced PBC research from several perspectives, including gut microbial composition, metabolite profiles, disease risk stratification, and transcriptomic alterations; however, these lines of evidence remain relatively fragmented. Specifically, liver differential expression analysis can identify genes associated with high-risk PBC, but it cannot determine whether these genes are related to gut microbiota-derived metabolites. Microbiome or metabolite studies may indicate the involvement of the gut-liver axis, but they do not directly identify host genes altered in high-risk PBC liver tissue. Disease-target databases can provide evidence of PBC relevance, yet they do not by themselves define the cell-type context of candidate targets [3–7].

Thus, a systematic integrative framework is still needed to address several related questions: which genes are significantly altered in high-risk PBC liver tissue, whether these genes have reported human microbial metabolite-host gene associations, and whether they have PBC disease-target evidence as well as interpretable expression patterns in the human liver cellular ecosystem. gutMGene, the Open Targets Platform, and the Human Liver Cell Atlas provide information on metabolite-host gene relationships, disease-target evidence, and human liver single-cell localization, respectively. In addition, external validation in independent PBC transcriptomic datasets is important for assessing whether prioritized candidate genes show disease-related expression patterns beyond the discovery dataset. However, systematic integration of these resources with high-risk PBC liver transcriptomic data and independent validation remains limited [8–10].

### Study aim

Based on this background, the present study aimed to establish a multi-database integrative framework for prioritizing candidate host targets associated with gut microbiota-derived metabolites in high-risk PBC. Specifically, differentially expressed genes between high-risk and low-risk PBC were first identified using the GSE79850 liver transcriptomic dataset. These results were then integrated with reported human microbial metabolite-host gene associations from gutMGene and PBC-associated targets from the Open Targets Platform to identify candidate genes supported by metabolite associations, transcriptomic alterations, and disease-target evidence. The Human Liver Cell Atlas was further used for reference cell-type localization of prioritized candidate genes. In addition, exploratory immune signature analysis was performed to provide supportive information on the immune-inflammatory transcriptional context of prioritized candidate genes, particularly CCL2. An external validation analysis using the GSE119600 whole-blood transcriptomic cohort was further conducted to assess whether the prioritized candidate genes showed PBC-related expression patterns in an independent dataset.

Importantly, this study was not designed to prove that gut microbiota-derived metabolites cause high-risk PBC or inadequate UDCA response. Rather, it aimed to provide a traceable and prioritized list of candidate targets for future experimental validation and independent cohort studies [8–10].

## Materials and methods

### Study design and data sources

This study was designed as a public database-based candidate target prioritization and external validation analysis. We integrated liver transcriptomic data, microbial metabolite-host gene associations, PBC disease-target evidence, human liver single-cell atlas information, and an independent blood transcriptomic cohort to identify and evaluate gut microbiota-derived metabolite-associated candidate host targets related to high-risk primary biliary cholangitis (PBC). The main data sources included GSE79850, gutMGene, the Open Targets Platform, the Human Liver Cell Atlas, and GSE119600. Detailed information on these datasets and resources is provided in Table 1 [8–10].

**Table 1:** Data sources used in this study.

Data source	Data type	Role in this study	Information used
**GSE79850**	PBC liver bulk transcriptome	Differential expression analysis	High Risk Group and Low Risk Group liver samples
**gutMGene**	Microbial metabolite-host gene associations	Identification of metabolite-associated host genes	Human microbial metabolite-host gene records
**Open Targets Platform**	Disease-target associations	Integration of PBC-associated genes	Gene symbols and globalScore
**Human Liver Cell Atlas**	Human liver single-cell atlas	Reference cell-type localization	Expression distribution of candidate genes across liver cell types
**GSE119600**	Whole-blood transcriptomic cohort	External validation	PBC and control blood transcriptomic samples

**Abbreviations:** PBC, primary biliary cholangitis; DEGs, differentially expressed genes; HLCA, Human Liver Cell Atlas.

### Differential expression analysis of GSE79850

The GSE79850 dataset from the Gene Expression Omnibus (GEO) database was used as the liver transcriptomic dataset for analyzing expression changes associated with high-risk PBC [3,11]. The expression matrix and sample annotation information of GSE79850 were downloaded using the GEOquery package in R [12]. This dataset included 24 liver tissue samples, consisting of 7 samples from the Low Risk Group, 9 samples from the High Risk Group, and 8 samples from the Control Group. Because the present study focused on expression alterations associated with the high-risk state of PBC, only High Risk Group and Low Risk Group samples were included in the subsequent differential expression analysis, while Control Group samples were not used in this comparison.

After extracting group information from the sample annotations, the Low Risk Group was set as the reference group and the High Risk Group as the comparison group. The final expression matrix used for differential expression analysis included 16 PBC samples, consisting of 9 High Risk Group samples and 7 Low Risk Group samples. Because the raw expression values showed a wide numerical range, suggesting that log transformation may not have been applied, the expression matrix was transformed using log2(expr + 1) before differential expression analysis.

Differential expression analysis was performed using the limma package in R [13]. After construction of the design matrix, a linear model was fitted to estimate expression differences between the High Risk Group and the Low Risk Group, and empirical Bayes moderation was applied to improve variance estimation. Differentially expressed genes were identified using the criteria of adjusted P value < 0.05 and |logFC| > 1. Multiple testing correction was performed using the Benjamini-Hochberg method [14]. In this study, logFC > 0 indicated higher expression in the High Risk Group, whereas logFC < 0 indicated higher expression in the Low Risk Group. The resulting differentially expressed genes were used for subsequent intersection analysis with gutMGene-derived microbial metabolite-host gene associations.

### Extraction of human microbial metabolite-host gene associations from gutMGene

The gutMGene database was used to obtain reported associations between gut microbial metabolites and host genes [8]. The file “Microbial metabolite-Host Gene.csv” was downloaded from gutMGene. This file contains information on microbial metabolites, host genes, species source, alteration direction, experimental methods, and supporting literature.

To ensure consistency with the human PBC transcriptomic data, only records annotated as human in the human/mouse field were retained. The main fields used in this study included Metabolite, Gene, Alteration, PMID, Associative mode, Experimental method, Condition, and Description. Among these fields, Metabolite indicated the name of the gut microbiota-related metabolite, Gene indicated the corresponding host gene, Alteration described the reported direction of the metabolite-associated effect on the host gene or related molecular process, and PMID was retained to trace the original supporting literature.

After filtering and curation, a total of 265 human microbial metabolite-host gene association records were obtained, involving 45 distinct metabolites and 154 host genes. The curated human gutMGene dataset was then intersected with the differentially expressed genes identified from the comparison between high-risk and low-risk PBC samples in GSE79850. This step was performed to identify candidate host genes supported by both gut microbiota-derived metabolite associations and differential expression evidence in high-risk PBC [3,8].

### Intersection between gutMGene host genes and GSE79850 DEGs

To identify candidate genes supported by both microbial metabolite-host gene associations and differential expression evidence in high-risk PBC, host genes from the curated human gutMGene dataset were intersected with significant differentially expressed genes from the GSE79850 high-risk PBC versus low-risk PBC comparison [3,8]. Specifically, the Gene field from the human microbial metabolite-host gene association records in gutMGene was used as the host gene list, while significant differentially expressed genes from GSE79850 were used as the transcriptomic evidence source. Genes present in both datasets were retained as candidate host genes.

For the intersected metabolite-gene association records, differential expression statistics from GSE79850, including logFC, P.Value, and adj.P.Val, were further merged. The expression direction of each candidate gene in high-risk PBC was determined according to logFC. Genes with logFC > 0 were defined as “Up in high-risk PBC”, whereas genes with logFC < 0 were defined as “Down in high-risk PBC”.

After intersection analysis, 15 gutMGene-derived metabolite-host gene candidate association records were obtained, involving 7 host genes: CCL2, MAPK3, CDKN1A, BCL2, G6PD, CD44, and FOS. These candidate records were then summarized at the gene level, including metabolite names, number of associated metabolites, alteration direction, PMID, differential expression statistics, and expression direction. The summarized results were used for subsequent integration with PBC disease-associated gene evidence.

### Integration with Open Targets PBC-associated genes

To further improve the disease relevance of the candidate genes in the context of PBC, disease-target association data from the Open Targets Platform were incorporated [9]. Because the standard disease term has been updated from the historical term “primary biliary cirrhosis” to “primary biliary cholangitis”, the Open Targets Platform was searched using the standard term “primary biliary cholangitis”. Disease association records corresponding to the historical synonym “primary biliary cirrhosis” were also included to improve retrieval completeness and maintain terminological consistency [1,2]. The associated targets data were exported in TSV format. After data import, gene symbols and globalScore values were extracted. The symbol field was used to represent target gene names, while globalScore was used as an integrated measure of disease-target association evidence in Open Targets [9].

After curation of the Open Targets data, records with missing gene symbols or symbols annotated as “No data” were removed. A total of 1,979 PBC-associated genes were retained. These PBC-associated genes were then intersected with the gutMGene ∩ GSE79850 DEG candidate metabolite-gene records to identify candidate targets supported by microbial metabolite-host gene associations, differential expression evidence in high-risk PBC, and PBC disease-target evidence. In this study, the three-way intersection was defined as follows:


$$\begin{array}{c} \text{GutMGenehumanhostgenes}\cap \text{GSE79850DEGs}\cap \text{OpenTargetsPBC}-\text{associatedgenes} \end{array}$$

Candidate genes included in the three-way intersection were required to meet all of the following criteria: first, the gene was present in human microbial metabolite-host gene association records from gutMGene; second, the gene met the criteria for significant differential expression in the GSE79850 high-risk PBC versus low-risk PBC comparison; and third, the gene had PBC disease-target association evidence in the Open Targets Platform. After three-way intersection analysis, 14 candidate metabolite-gene association records were retained, involving six core candidate genes: CCL2, CDKN1A, BCL2, CD44, FOS, and MAPK3. The results were then summarized at the gene level, including metabolite names, number of metabolites, alteration direction, PMID, logFC, P.Value, adjusted P value, expression direction, and Open Targets globalScore. These summarized data were used for subsequent candidate gene prioritization and visualization.

### Candidate gene prioritization

To support the ranking and interpretation of candidate genes from the three-way intersection, an exploratory priority score was constructed for the core candidate genes. This score incorporated the number of gut microbiota-related metabolites associated with each candidate gene, the magnitude of differential expression between high-risk and low-risk PBC samples, the statistical significance of differential expression, and the PBC disease-target association score from Open Targets. The formula was defined as follows:


$$\begin{array}{c} \text{priority}\_\text{score }=\text{ n}\_\text{metabolites }+\text{ abs}(\text{logFC})\;+\;-\text{log10}(\text{adj}.\text{P}.\text{Val})\;+\text{ globalScore} \end{array}$$

In this formula, n_metabolites represents the number of distinct metabolites associated with each candidate gene; abs(logFC) represents the absolute magnitude of expression difference between high-risk and low-risk PBC samples; -log10(adj.P.Val) represents the statistical significance of differential expression; and globalScore represents the integrated disease-target association score between the gene and PBC in the Open Targets Platform [9].

The priority_score was calculated for each core candidate gene, and genes were ranked in descending order according to this score. This ranking was used to identify candidate targets that may deserve greater attention in subsequent interpretation and experimental validation. It should be noted that the priority_score constructed in this study was exploratory and was used only for relative prioritization among candidate genes. It should not be interpreted as a true biological effect size, causal disease strength, or clinical predictive performance.

In addition, the scoring strategy intentionally gave weight to the breadth of functional connectivity between candidate genes and upstream gut microbiota-related metabolites, as represented by n_metabolites. Therefore, the absolute values of the priority score should be interpreted as conceptual guideposts for empirical validation ranking rather than mathematically normalized biological effect sizes. In other words, the purpose of this score was not to precisely quantify the equal contribution of different evidence sources, but to help identify candidate targets that may be more suitable for further validation within a limited candidate gene set. The actual biological functions and mechanisms of these candidate genes require further experimental investigation.

### Human Liver Cell Atlas-based reference cell-type localization

To further examine the reference cell-type distribution of the three-way candidate genes across major human liver cell populations, we performed cell-type localization analysis using the Human Liver Cell Atlas module of the Liver Cell Atlas [10]. Because publicly available PBC-specific human liver single-cell datasets with comprehensive cell-type annotations remain limited, this atlas was used as a reference resource to characterize the baseline cellular localization patterns of candidate genes.

The queried genes were the six core candidate genes identified from the three-way intersection, including CCL2, CDKN1A, BCL2, CD44, FOS, and MAPK3. In the All Liver Cells module of the Human Liver Atlas, each gene symbol was entered separately to obtain expression UMAP plots and cell-type localization information. The number of expressing cells, the proportion of expressing cells, and the major expression distribution patterns were recorded for each candidate gene. Representative CCL2 localization results were presented in the main manuscript, while the localization patterns of the remaining five candidate genes were provided in S1 Fig. The processed HLCA objects used to reproduce the candidate-gene localization summaries are provided in S1 Data.

This analysis was performed to characterize the reference cell-type localization patterns of core candidate genes across human liver cell populations and to provide supportive information regarding the potential cellular context of these candidate genes. HLCA-based analyses were interpreted as reference cell-type localization analyses rather than disease-specific expression analyses. Single-cell visualization and expression pattern visualization were performed based on the Seurat framework (version 5.5.1).

### Exploratory immune signature analysis

To further explore the immune-inflammatory context of the core candidate genes, an exploratory immune signature analysis was performed using the GSE79850 high-risk and low-risk PBC samples. The log2(expr + 1)-transformed expression matrix used for differential expression analysis was also used for this analysis.

Immune-related marker gene sets were manually curated based on established immune cell markers and previously reported immune-related signatures from the literature, representing monocyte/macrophage-related features, T cells, B cells, NK cells, inflammatory response, and chemokine signaling. The monocyte/macrophage-related signature included CD14, LYZ, FCGR3A, MS4A7, C1QA, C1QB, C1QC, CD68, and ITGAM. The T-cell signature included CD3D, CD3E, CD2, TRAC, TRBC1, and TRBC2. The B-cell signature included MS4A1, CD79A, CD79B, and CD19. The NK-cell signature included NKG7, GNLY, KLRD1, PRF1, and GZMB. The inflammatory response signature included CCL2, CXCL8, IL1B, TNF, CXCL10, CCL5, NFKB1, and STAT1. The chemokine signaling signature included CCL2, CCL5, CXCL8, CXCL10, CCR1, CCR2, CCR5, and CXCR4. The complete immune signature marker gene lists are provided in S1 Table.

For each immune-related signature, only marker genes present in the GSE79850 expression matrix were used. For each sample, the signature score was calculated as the average expression value of the available marker genes in the corresponding signature. This approach was selected because the present analysis was designed as an exploratory assessment of predefined immune-related transcriptional patterns rather than a comprehensive pathway enrichment analysis. Given the limited sample size and the predefined biological interpretation of candidate immune signatures, simple averaged expression scoring was used to provide an intuitive and reproducible estimation of immune-related transcriptional activity. Differences in signature scores between high-risk and low-risk PBC samples were compared using the Wilcoxon rank-sum test. Spearman correlation analysis was used to evaluate the relationship between CCL2 expression and immune-related signature scores. Multiple testing correction was performed using the Benjamini-Hochberg method when multiple immune signatures were compared. This analysis was considered exploratory and was used only to provide supportive information on the immune-inflammatory transcriptional context of prioritized candidate genes.

### External validation using GSE119600

To further assess whether the core candidate genes showed disease-related expression patterns in an independent PBC dataset, external validation was performed using the GSE119600 whole-blood transcriptomic cohort. The expression matrix and sample annotation information of GSE119600 were downloaded from the GEO database using the GEOquery package in R. This dataset contains whole-blood transcriptomic profiles from patients with primary biliary cholangitis, primary sclerosing cholangitis, Crohn’s disease, ulcerative colitis, and healthy controls. In the present validation analysis, only PBC samples and control samples were retained, including 90 PBC samples and 47 control samples.

Because GSE119600 is a microarray dataset, probe identifiers were mapped to gene symbols according to the platform annotation. Probes without gene symbols were removed. When multiple probes mapped to the same gene symbol, the average expression value of these probes was used to generate a gene-level expression matrix. The expression levels of the six core candidate genes identified from the three-way integration analysis, including CCL2, CDKN1A, BCL2, CD44, FOS, and MAPK3, were extracted from the gene-level expression matrix.

Differences in candidate gene expression between PBC samples and control samples were assessed using the Wilcoxon rank-sum test. Multiple testing correction was performed using the Benjamini-Hochberg method. This external validation analysis was used to evaluate whether the prioritized candidate genes showed PBC-related expression patterns in an independent blood transcriptomic cohort. Because GSE119600 is a blood-based dataset and does not provide high-risk versus low-risk PBC stratification, this analysis was not considered a direct validation of high-risk PBC-specific liver expression changes.

### Visualization and statistical analysis

Data processing, statistical analysis, and visualization were performed using R version 4.5.0. GEO data were obtained and processed using GEOquery version 2.78.0, and differential expression analysis was conducted using limma version 3.66.0 [12,13]. Multiple testing correction was performed using the Benjamini–Hochberg method [14]. Data manipulation and visualization were performed using tidyverse version 2.0.0, data.table version 1.18.4, ggplot2 version 4.0.3, pheatmap version 1.0.13, ggrepel version 0.9.8, patchwork version 1.3.2, readxl version 1.4.5, writexl version 1.5.4, and openxlsx version 4.2.8.1; ggplot2-based visualizations followed the grammar-of-graphics framework [15]. Network visualization was conducted using igraph version 2.3.1, tidygraph version 1.3.1, and ggraph version 2.2.2 [16].

Differential expression results from GSE79850 were visualized using a volcano plot, with logFC on the x-axis and -log10(adjusted P value) on the y-axis. Genes were categorized as upregulated in high-risk PBC, downregulated in high-risk PBC, or not significantly different according to the thresholds of adjusted P value < 0.05 and |logFC| > 1. To display the expression patterns of candidate genes between high-risk and low-risk PBC samples, heatmaps and boxplots were generated using the log2(expr + 1)-transformed expression matrix. Heatmaps were produced using the pheatmap package, and expression values were scaled by gene to show relative expression differences across samples. Boxplots were generated using the ggplot2 package to show the expression distribution of each candidate gene between the high-risk PBC and low-risk PBC groups [15].

To visualize the associations between gut microbiota-related metabolites and candidate host genes, a metabolite-gene network was constructed. Nodes in the network represented metabolites and genes, while edges represented reported microbial metabolite-host gene associations in gutMGene. Edge types were distinguished according to the Alteration field. The network was generated using the igraph, tidygraph, and ggraph packages, with a force-directed layout [16]. For the core candidate genes identified from the three-way intersection, a candidate gene priority ranking bar plot was further generated using the priority_score as the ranking variable. This plot was used to display the relative priority of different candidate genes within the integrative screening framework of this study.

All result tables were saved in CSV or Excel format. Continuous expression data were transformed using log2(expr + 1) before differential expression analysis and immune signature scoring. Multiple testing correction was performed using the Benjamini-Hochberg method when applicable [14]. Immune signature comparison plots and correlation plots were generated using ggplot2 and combined using patchwork. Unless otherwise specified, all analyses in this study were exploratory integrative analyses. The results were intended for candidate target screening, relative prioritization, and hypothesis generation for future experimental validation, and were not intended to directly prove disease-causal mechanisms.

### Ethics statement

This study was based entirely on publicly available and de-identified datasets and online resources, including GSE79850, GSE119600, gutMGene, the Open Targets Platform, and the Human Liver Cell Atlas. No new human participants were recruited, no new human tissue or blood samples were collected, and no animal experiments were performed. Therefore, additional ethics committee approval and informed consent were not required.

## Results

### Differentially expressed genes in high-risk PBC liver samples

We first analyzed gene expression differences between high-risk and low-risk PBC liver tissue samples using the GSE79850 dataset. This dataset contained 24 liver tissue samples, including 7 samples from the Low Risk Group, 9 samples from the High Risk Group, and 8 samples from the Control Group. Because this study focused on transcriptomic alterations associated with the high-risk state of PBC, only High Risk Group and Low Risk Group samples were included in the subsequent analysis, while Control Group samples were not included in this comparison.

**Fig 1:**
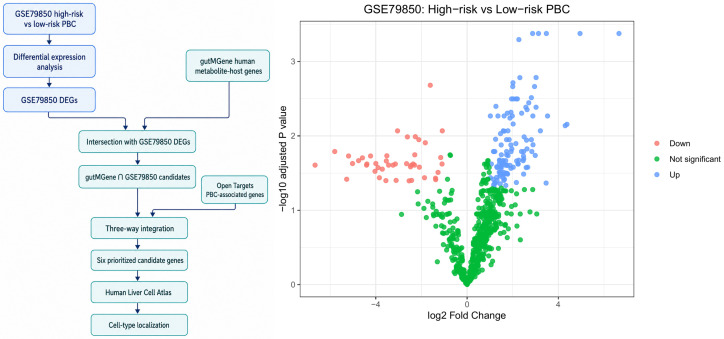
Study workflow and differential expression analysis of GSE79850. (A) Workflow of the integrative analysis. Differentially expressed genes were first identified between high-risk and low-risk PBC liver samples in GSE79850. Candidate genes were then screened by integrating gutMGene human metabolite-host gene associations and Open Targets PBC-associated genes. Human Liver Cell Atlas-based reference cell-type localization, exploratory immune signature analysis, and GSE119600 external validation were further performed to support the interpretation and validation of prioritized candidate genes. (B) Volcano plot showing differentially expressed genes between high-risk and low-risk PBC liver samples in GSE79850. Red and blue dots indicate significantly downregulated and upregulated genes, respectively, while green dots indicate genes without significant differential expression.

After log2(expr + 1) transformation of the expression matrix, differential expression analysis between high-risk and low-risk PBC samples was performed using the limma package. Using adjusted P value < 0.05 and |logFC| > 1 as the screening criteria, a total of 163 significant differentially expressed genes were identified. Among them, 119 genes were upregulated and 44 genes were downregulated in the high-risk PBC group. The summary counts of each analytical step are shown in Table 2. The overall study workflow and the volcano plot of differentially expressed genes are shown in Fig 1.

**Table 2:** Summary counts of each analytical step.

Analytical_step	Count
GSE79850 high-risk vs low-risk DEGs	163
Upregulated DEGs in high-risk PBC	119
Downregulated DEGs in high-risk PBC	44
gutMGene human metabolite-host gene records	265
gutMGene human host genes	154
gutMGene ∩ GSE79850 DEG records	15
gutMGene ∩ GSE79850 DEG unique genes	7
Open Targets PBC-associated genes	1,979
Three-way candidate metabolite-gene records	14
Three-way unique candidate genes	6

**Note:** DEG, differentially expressed gene; PBC, primary biliary cholangitis. Counts represent the number of genes or records retained at each step of the integrative analysis workflow.

### gutMGene-derived metabolite-host gene candidates overlapping with GSE79850 DEGs

To further identify gut microbiota-derived metabolite-associated host targets potentially related to the high-risk state of PBC, we curated human microbial metabolite-host gene association records from the gutMGene database. After filtering for human records, a total of 265 metabolite-host gene association records were obtained, involving 45 metabolites and 154 host genes. Human host genes from gutMGene were then intersected with significant differentially expressed genes from the GSE79850 high-risk PBC versus low-risk PBC comparison.

This intersection yielded 15 candidate metabolite-gene association records involving 7 host genes, including CCL2, MAPK3, CDKN1A, BCL2, G6PD, CD44, and FOS. The gut microbiota-related metabolites linked to these candidate genes included acetate, butyrate, propionate, succinate, urolithin A, trimethylamine oxide, and hydroquinone. Among these candidates, CCL2 was connected to the largest number of metabolites, including acetate, butyrate, propionate, and succinate. MAPK3 was associated with metabolites such as succinate, CD44 was associated with trimethylamine oxide, and FOS was associated with butyrate.

**Fig 2:**
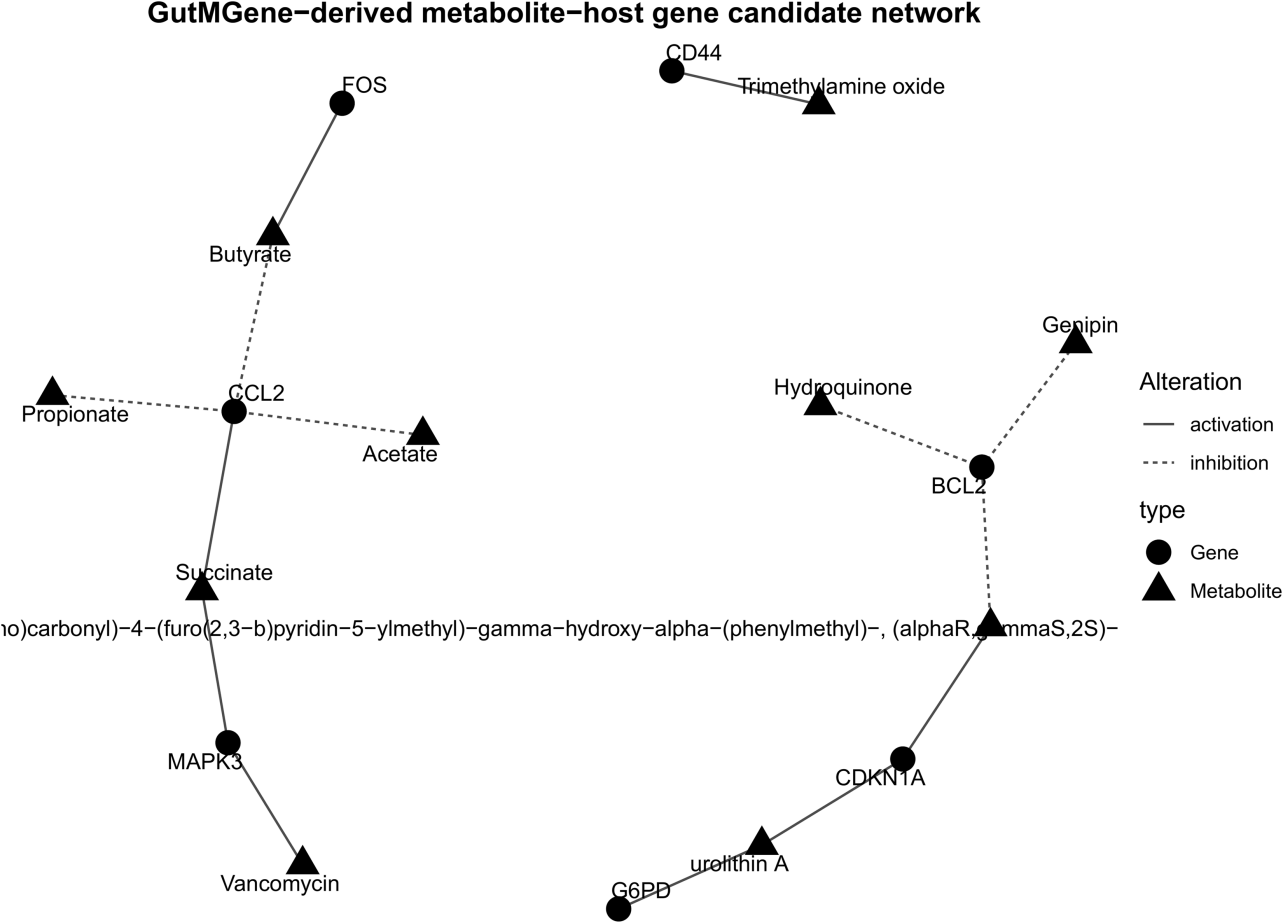
gutMGene-derived metabolite-host gene candidates overlapping with GSE79850 DEGs. The network shows candidate microbial metabolite-host gene associations identified by intersecting gutMGene human metabolite-host genes with differentially expressed genes from GSE79850. Nodes represent metabolites or host genes, and edges represent reported metabolite-gene associations in gutMGene.

These findings indicate that a subset of high-risk PBC-related differentially expressed genes also had reported gut microbiota-derived metabolite association evidence. These genes were therefore retained as candidate host targets for subsequent three-way integration analysis. The gutMGene-derived metabolite-host gene candidate network is shown in Fig 2.

### Three-way integration identifies six PBC-related candidate genes

To further strengthen the disease-context relevance of the candidate genes, PBC-associated genes from the Open Targets Platform were incorporated. After data curation, a total of 1,979 disease-associated target genes related to primary biliary cholangitis were obtained. We then performed a three-way intersection among gutMGene human host genes, significant DEGs from the GSE79850 high-risk versus low-risk PBC comparison, and Open Targets PBC-associated genes.

The three-way intersection identified 14 candidate metabolite-gene association records involving six core candidate genes: CCL2, CDKN1A, BCL2, CD44, FOS, and MAPK3. Compared with the 7 candidate genes obtained from the gutMGene ∩ GSE79850 intersection, G6PD was not retained among the final core candidate genes because it was not included in the Open Targets PBC-associated gene intersection. All six core candidate genes were upregulated in the high-risk PBC group, suggesting that they may be related to transcriptional activation in the high-risk PBC state.

**Fig 3:**
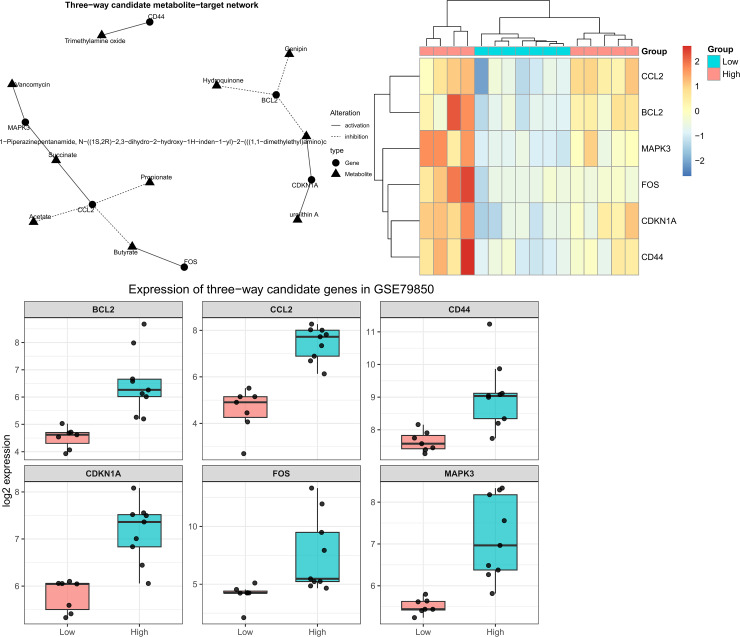
Three-way integration of gutMGene, GSE79850 DEGs, and Open Targets PBC-associated genes. (A) Network of three-way candidate metabolite-gene associations. (B) Heatmap showing expression patterns of the six core candidate genes in high-risk and low-risk PBC samples. (C) Boxplots showing expression differences of the six core candidate genes between high-risk and low-risk PBC samples. Differential expression analysis was performed between high-risk (n = 9) and low-risk (n = 7) PBC liver samples from the GSE79850 dataset, with control samples (n = 8) excluded from the high-risk versus low-risk comparison. Expression values in the heatmap were scaled by gene using row-wise Z-score transformation.

Among the three-way candidate genes, CCL2 remained the candidate gene connected to multiple gut microbiota-related metabolites, including acetate, butyrate, propionate, and succinate. CDKN1A, BCL2, CD44, FOS, and MAPK3 also had reported associations with different metabolites. The three-way intersection network, heatmap, and boxplots of the six core candidate genes are shown in Fig 3.

### Priority ranking of candidate genes

To compare the relative priority of candidate genes from the three-way intersection, we constructed an exploratory priority score based on the number of metabolite connections, the magnitude of differential expression, the statistical significance of differential expression, and the Open Targets disease association score. This score was used to rank the six core candidate genes and to help identify candidate targets that may deserve greater attention in subsequent studies. The ranked three-way candidate genes and their priority scores are summarized in Table 3.

**Table 3:** Ranked three-way candidate genes.

Gene	main_metabolites	n_metabolites	logFC	adj.P.Val	globalScore	priority_score
**CCL2**	Acetate; Butyrate; Propionate; Succinate	4	2.869	0.00042	0.0810	10.327
**BCL2**	Long-chain compound; Genipin; Hydroquinone	3	2.017	0.00692	0.0362	7.213
**FOS**	Butyrate	1	3.461	0.04310	0.0081	5.835
**MAPK3**	Succinate; Vancomycin	2	1.630	0.00863	0.0037	5.697
**CDKN1A**	Long-chain compound; Urolithin A	2	1.351	0.00540	0.0492	5.668
**CD44**	Trimethylamine oxide	1	1.424	0.02150	0.0136	4.104

**Note:** The priority score was calculated as:

priority score = n_metabolites + |logFC| −log10(adj.P.Val) + globalScore.

This score was used for exploratory prioritization of candidate genes and does not represent causal strength or biological effect size.

**Abbreviations:** logFC, logarithmic fold change; adj.P.Val, adjusted P value.

**Fig 4:**
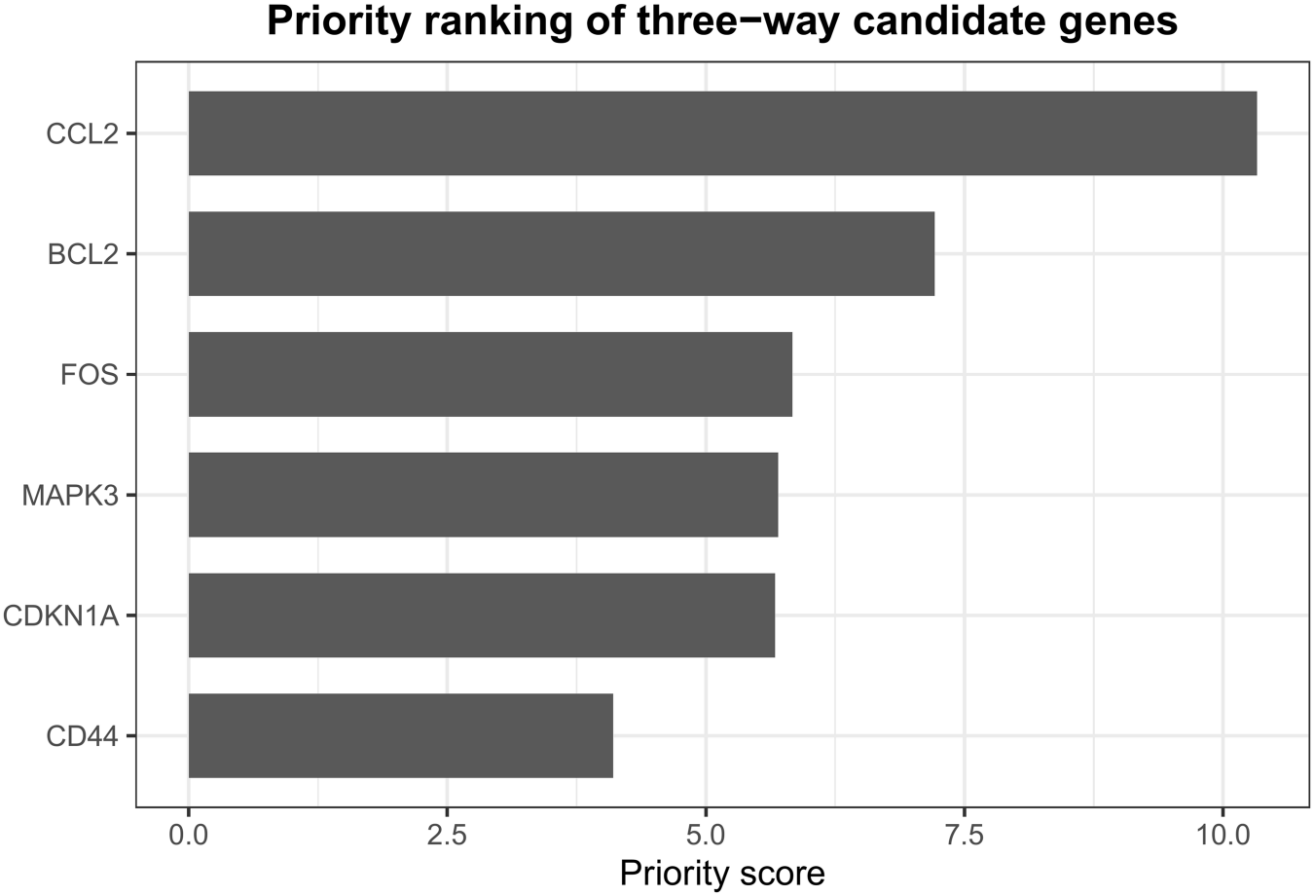
Priority ranking of three-way candidate genes. Candidate genes were ranked using an exploratory priority score integrating the number of connected metabolites, absolute logFC, statistical significance, and Open Targets globalScore. The ranking was used only for relative prioritization and does not represent causal strength or biological effect size. In addition to CCL2, CDKN1A, BCL2, CD44, FOS, and MAPK3 were also retained in the three-way intersection analysis and showed upregulated expression in the high-risk PBC group. It should be noted that the priority score constructed in this study was used only for exploratory ranking of candidate genes and does not represent a true biological effect size or causal disease strength. These candidate genes require further validation in independent cohorts and experimental systems.

The priority ranking showed that CCL2 was the top-ranked candidate within the discovery integrative framework based on GSE79850, gutMGene, and Open Targets evidence. The priority ranking of the six core candidate genes is shown in Fig 4. CCL2 was not only significantly upregulated in the GSE79850 high-risk PBC group, but was also linked to multiple reported gut microbiota-related metabolites, including acetate, butyrate, propionate, and succinate. Based on these discovery results, CCL2 may represent a candidate node linking reported gut microbiota-derived metabolite associations with transcriptomic alterations in high-risk PBC liver tissue.

### Cell-type localization in the Human Liver Cell Atlas

To further characterize the reference cell-type localization patterns of the core candidate genes across major human liver cell populations, we queried the All Liver Cells module of the Human Liver Cell Atlas. Overall, the six core candidate genes showed heterogeneous reference localization patterns in the Human Liver Cell Atlas, with CCL2 displaying a relatively restricted localization pattern in immune-related cell populations.

**Fig 5:**
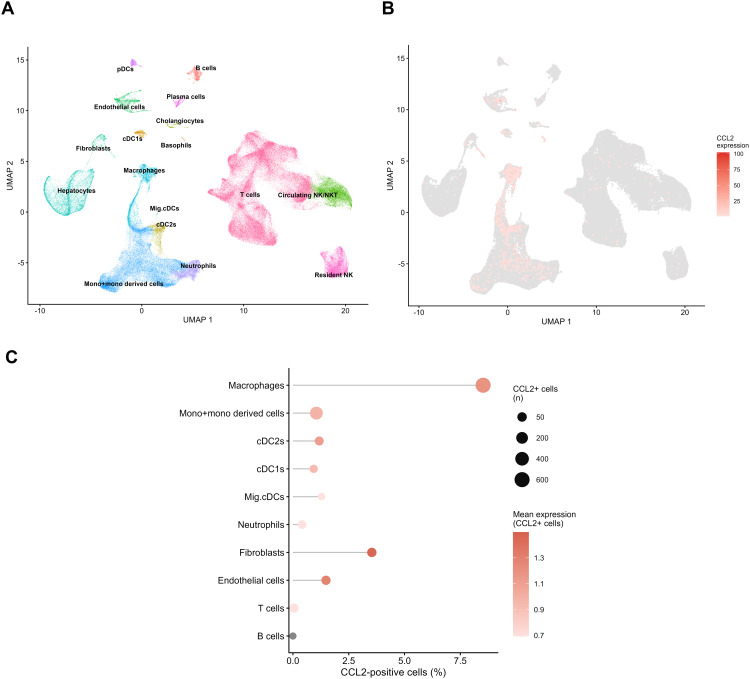
Single-cell localization analysis of CCL2 expression patterns in the Human Liver Cell Atlas. (A) Cell type annotation of the Human Liver Cell Atlas showing major liver cell populations. (B) UMAP visualization of CCL2 expression across individual cells. Red indicates higher expression levels, whereas gray indicates low or undetectable expression. (C) Distribution of CCL2-positive cells across major liver cell populations. The x-axis represents the proportion of CCL2-positive cells within each cell population. Dot size represents the number of CCL2-positive cells, and color intensity indicates the mean expression level among CCL2-positive cells.

Among them, CCL2 showed a relatively restricted expression pattern. CCL2 expression was detected in 1,166 cells, accounting for 0.7% of all cells. In terms of cell-type distribution, CCL2 was mainly detected in subsets of immune-related cell populations, including macrophages and monocyte-derived cells, suggesting a more immune-inflammatory cell-type context within the liver. Notably, this highly restricted and cell-type-biased baseline expression pattern does not diminish its potential relevance as a candidate gene. Instead, when considered together with its marked upregulation in high-risk PBC liver tissue, this pattern suggests that CCL2 shows preferential localization within specific hepatic cellular environments in the reference human liver atlas rather than representing a nonspecific marker of global cellular stress. The cell-type localization pattern of CCL2 in the Human Liver Cell Atlas is shown in Fig 5.

In contrast, CD44 and FOS showed broader expression patterns in the Human Liver Cell Atlas. CD44 expression was detected in 69,298 cells, accounting for 41.3% of all cells, while FOS expression was detected in 107,326 cells, accounting for 64.0% of all cells. These findings indicate that CD44 and FOS exhibited relatively widespread expression patterns across liver cell populations, potentially reflecting their involvement in broader biological processes such as inflammation, cell adhesion, or cellular stress responses. CDKN1A, BCL2, and MAPK3 showed lower expression proportions, with expression detected in 15,982 cells (9.5%), 22,321 cells (13.3%), and 7,526 cells (4.5%), respectively. The reference cell-type localization patterns of these five candidate genes are shown in S1 Fig.

It should be noted that the Human Liver Cell Atlas was used in this study as a reference human liver single-cell resource [10] to characterize the baseline cell-type localization of candidate genes across major human liver cell types, rather than as a PBC-specific single-cell cohort.

### Exploratory immune signature analysis

To further explore the immune-inflammatory context of the core candidate genes in high-risk PBC, we performed an exploratory marker gene-based immune signature analysis using the GSE79850 high-risk and low-risk PBC samples. Immune-related signature scores were calculated based on the average expression of marker genes representing monocyte/macrophage-related features, T cells, B cells, NK cells, inflammatory response, and chemokine signaling.

Compared with the low-risk PBC group, the high-risk PBC group showed a higher chemokine signaling signature score (P = 0.041), while the inflammatory response signature showed an increasing trend (P = 0.072). Other immune-related signatures, including T-cell, B-cell, and NK-cell signatures, also tended to be higher in high-risk PBC samples, but these differences did not reach statistical significance. After multiple testing correction, none of the immune signature comparisons remained statistically significant.

**Fig 6:**
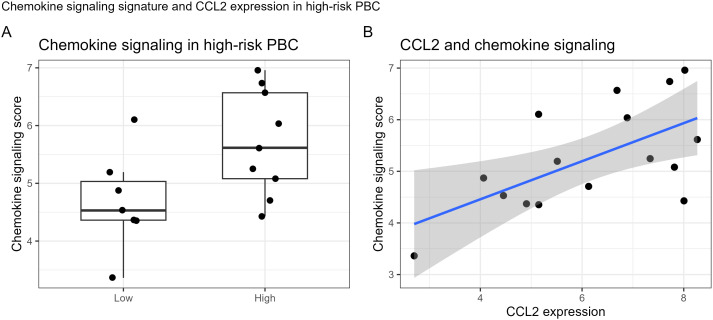
Exploratory immune signature analysis in high-risk primary biliary cholangitis. (A) Comparison of chemokine signaling signature scores between low-risk and high-risk PBC samples in the GSE79850 dataset. (B) Correlation between CCL2 expression and chemokine signaling signature score in the GSE79850 dataset.

Spearman correlation analysis further showed that CCL2 expression was positively correlated with the chemokine signaling signature score (Spearman r = 0.547, P = 0.028) and showed a positive trend with the inflammatory response signature score (Spearman r = 0.462, P = 0.072). In contrast, CCL2 expression was not clearly correlated with the monocyte/macrophage signature in this dataset. These findings provide exploratory support for a chemokine-related immune-inflammatory transcriptional context of CCL2 in high-risk PBC, but they should not be interpreted as definitive evidence of immune cell infiltration or causal immune activation. The comparison of chemokine signaling signature scores and the correlation between CCL2 expression and chemokine signaling are shown in Fig 6.

### External validation in the GSE119600 blood transcriptomic cohort

To further assess whether the core candidate genes showed disease-related expression patterns in an independent PBC dataset, we performed an external validation analysis using the GSE119600 whole-blood transcriptomic cohort. This validation analysis included 90 PBC samples and 47 control samples. Because GSE119600 is a blood-based cohort rather than a liver tissue dataset and does not provide high-risk versus low-risk PBC stratification, this analysis was used to evaluate whether the prioritized candidate genes showed general PBC-related expression differences in an independent dataset, rather than to validate high-risk PBC-specific hepatic expression changes.

Among the six core candidate genes, CD44, FOS, and CDKN1A were significantly upregulated in PBC samples compared with controls after multiple testing correction. Specifically, CD44 showed higher expression in PBC than in controls (mean expression: 2918.38 vs. 2327.67; P = 3.45 × 10^-5; FDR = 2.07 × 10^-4). FOS was also significantly upregulated in PBC samples (mean expression: 997.58 vs. 729.26; P = 2.60 × 10^-4; FDR = 7.80 × 10^-4). CDKN1A showed a moderate but statistically significant increase in PBC samples (mean expression: 583.19 vs. 528.20; P = 0.0127; FDR = 0.0254). MAPK3 showed a higher mean expression in PBC samples than in controls, but this difference did not reach statistical significance. In contrast, CCL2 and BCL2 did not show consistent upregulation in this blood transcriptomic dataset.

**Fig 7:**
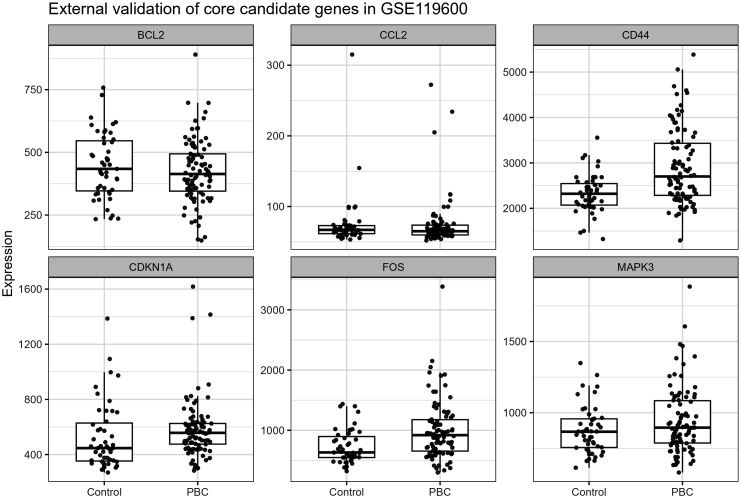
External validation of core candidate genes in the GSE119600 blood transcriptomic cohort. Expression distributions of the six core candidate genes, including CCL2, CDKN1A, BCL2, CD44, FOS, and MAPK3, were compared between PBC samples and control samples in the GSE119600 dataset. CD44, FOS, and CDKN1A showed significantly higher expression in PBC samples, whereas CCL2 and BCL2 did not show consistent upregulation in this blood-based validation cohort.

These external validation results indicate that part of the prioritized candidate gene set, particularly CD44, FOS, and CDKN1A, showed independent disease-related expression evidence in PBC. However, the lack of consistent CCL2 upregulation in the blood cohort indicates that CCL2 was not externally validated as a peripheral blood expression marker in GSE119600. This discrepancy suggests that CCL2 may be more closely related to the local hepatic microenvironment or high-risk liver tissue context, but this interpretation requires further validation in independent liver tissue cohorts or PBC-specific spatial and single-cell datasets. The expression distributions of the six core candidate genes in the GSE119600 validation cohort are shown in Fig 7.

## Discussion

### Main findings

This study focused on gut microbiota-derived metabolite-associated candidate host targets related to high-risk primary biliary cholangitis (PBC). We integrated liver transcriptomic data, gutMGene-derived metabolite-host gene associations, Open Targets disease-target evidence, and Human Liver Cell Atlas-based reference cell-type localization information [3,8–10]. Unlike analyses that rank genes solely according to differential expression, this study incorporated multiple layers of evidence into a single screening framework. These layers included whether candidate genes were differentially expressed in high-risk PBC liver tissue, whether they had reported microbial metabolite-host gene associations, whether they had PBC disease-target evidence, and whether they showed interpretable expression localization in the human liver cell atlas. Based on this strategy, six core candidate genes were identified from the three-way intersection, namely CCL2, CDKN1A, BCL2, CD44, FOS, and MAPK3.

The original GSE79850 study suggested that high-risk and low-risk PBC patients already showed distinct hepatic molecular features at the early diagnostic stage, and that high-risk disease was associated with subsequent inadequate UDCA response and risk of disease progression [3]. Building on this transcriptomic background, the present study further incorporated gut microbiota-derived metabolite association evidence and disease-target evidence, which helped refine the interpretation of candidate molecular networks related to high-risk PBC [8–9].

Among all candidate genes, CCL2 showed the most convergent evidence within the discovery integrative framework based on GSE79850, gutMGene, Open Targets, and Human Liver Cell Atlas information. It was significantly upregulated in the high-risk PBC liver samples, had reported associations with multiple gut microbiota-related metabolites, including acetate, butyrate, propionate, and succinate, showed PBC disease-target evidence in Open Targets, and displayed a relatively restricted baseline expression distribution biased toward immune-related cell populations in the human liver single-cell atlas [8–10]. In addition, exploratory immune signature analysis showed that high-risk PBC samples had a higher chemokine signaling signature score, and CCL2 expression was positively correlated with the chemokine signaling signature. These findings support the interpretation of CCL2 as a chemokine-related hepatic microenvironment-associated candidate node in high-risk PBC.

However, the external validation analysis using the GSE119600 whole-blood transcriptomic cohort provided a more nuanced picture. CD44, FOS, and CDKN1A were significantly upregulated in PBC samples compared with controls, whereas CCL2 did not show consistent upregulation in this blood-based dataset. Therefore, the external validation results support the disease-related expression relevance of part of the prioritized candidate gene set, particularly CD44, FOS, and CDKN1A, while suggesting that CCL2 may be more closely related to the local hepatic high-risk microenvironment rather than a broadly detectable peripheral blood expression marker.

### CCL2 as a key immune-related candidate target

CCL2 encodes monocyte chemoattractant protein-1 and is one of the core ligands in the CCL2/CCR2 chemokine axis. It is commonly associated with monocyte recruitment, macrophage infiltration, and inflammatory tissue remodeling [17]. For PBC, a disease characterized by bile duct injury, intrahepatic immune activation, and chronic inflammation, CCL2 upregulation should not be viewed merely as an isolated differential expression event. A more reasonable interpretation is that it may reflect alterations in intrahepatic immune cell recruitment and the inflammatory microenvironment in the high-risk state.

Reuveni et al. reported that CCR2-positive monocytes played an important role in the process of cholangitis in a model of autoimmune cholangitis, and that CCR2 deficiency or pharmacological inhibition alleviated experimental autoimmune cholangitis. This finding provides independent biological support for considering CCL2 as an immune-related candidate node in the present study [17].

In this study, CCL2 was linked to acetate, butyrate, propionate, and succinate, suggesting that it may lie at an intersection between gut microbiota-related metabolic signals and intrahepatic immune-inflammatory responses. According to the alteration directions recorded in gutMGene, the relationships between CCL2 and different metabolites did not suggest a simple unidirectional linear pattern, but instead showed a network with both activation and inhibition annotations [8]. For example, records related to short-chain fatty acids more often suggested potential involvement in the suppression of inflammation-related host responses, whereas records related to succinate appeared closer to a pro-inflammatory or activating signal context [6–8]. This multidirectional pattern suggests that CCL2 may be better understood as a candidate immune node receiving convergent upstream metabolic signals, rather than as a target driven by a single metabolite.

However, this type of “connection” does not mean that this study proved direct regulation of CCL2 by these metabolites in patients with PBC. More precisely, the present study shows that CCL2 has reported evidence in multiple microbial metabolite-host gene associations recorded in existing literature-curated databases. gutMGene is a manually curated database of microbe, metabolite, and host gene relationships, with each record derived from published experimental or literature evidence. It is suitable for candidate target screening, but it cannot replace metabolite measurements in disease cohorts or functional experimental validation [8]. Therefore, the interpretation of CCL2 in this study should be limited to a candidate connecting node supported by convergent integrative evidence, rather than a proven pathogenic mechanism.

The cell-type localization of CCL2 further improves its interpretability as a prioritized candidate target. The Human Liver Cell Atlas showed that CCL2 was not broadly expressed across all liver cell types, but was detected only in a small proportion of cells and showed a baseline localization tendency toward immune-related populations such as macrophages and monocyte-derived cells. These observations should be interpreted as reference cell-type localization information in normal human liver rather than direct evidence of disease-specific cellular sources in high-risk PBC. Nevertheless, the immune-related localization tendency of CCL2 in the reference atlas is biologically compatible with its known chemokine function and provides supportive context for interpreting the hepatic transcriptomic findings observed in high-risk PBC [10,17].

The exploratory immune signature analysis in GSE79850 further supported this interpretation. High-risk PBC samples showed a higher chemokine signaling signature score than low-risk samples, while the inflammatory response signature showed an increasing trend. Moreover, CCL2 expression was positively correlated with the chemokine signaling signature and showed a positive trend with the inflammatory response signature. Although these findings should be interpreted cautiously because of the small sample size and exploratory nature of the analysis, they provide additional transcriptomic support for placing CCL2 in a chemokine-related immune-inflammatory context in high-risk PBC.

The GSE119600 external validation results should also be considered when interpreting CCL2. In this independent whole-blood cohort, CCL2 did not show consistent upregulation in PBC compared with controls. This finding does not necessarily contradict the liver-based discovery results, because blood transcriptomic profiles may not capture local hepatic immune-inflammatory signals. In addition, the discovery and validation cohorts addressed different biological questions, with GSE79850 comparing high-risk and low-risk PBC liver tissues, whereas GSE119600 compared patients with PBC and healthy controls in peripheral blood. Therefore, the absence of consistent CCL2 upregulation in GSE119600 may reflect differences in tissue source, disease stratification, or both. However, it indicates that CCL2 should not be interpreted as an externally validated peripheral blood biomarker in this study. Instead, CCL2 is better positioned as a hepatic microenvironment-associated candidate node that requires further validation in independent liver tissue cohorts, PBC-specific single-cell datasets, or spatial transcriptomic studies.

### CD44, FOS, and CDKN1A as externally supported candidate genes

In addition to CCL2, CD44, FOS, and CDKN1A deserve attention because they were not only retained in the three-way integration analysis but also showed independent disease-related expression support in the GSE119600 whole-blood transcriptomic cohort. In the external validation analysis, these three genes were significantly upregulated in PBC samples compared with controls after multiple testing correction. This finding suggests that part of the prioritized candidate gene set may represent PBC-related transcriptional signals that are detectable beyond the discovery liver transcriptomic dataset.

CD44 is a cell adhesion-related molecule involved in hyaluronan binding, cell migration, inflammatory cell recruitment, and tissue remodeling [18,19]. Previous bioinformatic studies of PBC have also identified CD44 as a candidate gene, suggesting that it may be related to immune-associated transcriptional changes in PBC [18]. In the present study, CD44 entered the three-way intersection, was upregulated in high-risk PBC liver samples, showed a broad expression distribution in the Human Liver Cell Atlas, and was significantly upregulated in the independent GSE119600 blood cohort. Taken together, these findings suggest that CD44 may reflect adhesion, immune activation, and tissue response programs involving multiple cellular compartments.

FOS is a classical immediate early gene and is commonly regarded as an indicator of cellular activation, stress responses, and rapid transcriptional responses [20]. In this study, FOS was upregulated in high-risk PBC liver samples, showed a broad expression pattern in the human liver atlas, and was also significantly upregulated in the GSE119600 validation cohort. These findings suggest that FOS may represent a broadly distributed inflammatory or stress-activation-related transcriptional signal in PBC. Therefore, FOS should be interpreted as a general transcriptional activation-related candidate rather than a specific terminal target of gut microbiota-derived metabolite effects.

CDKN1A, which is involved in cell cycle regulation and stress-related transcriptional responses, was also retained in the three-way integration analysis and showed significant upregulation in the GSE119600 validation cohort. This result provides additional support for its disease-related expression relevance in PBC. However, as with CD44 and FOS, the current findings do not prove a directional functional role for CDKN1A in PBC pathogenesis. Rather, they indicate that CDKN1A is part of a prioritized candidate gene set with multilayer database evidence and independent blood transcriptomic support.

BCL2 and MAPK3 were also retained in the three-way integration analysis. However, they did not show statistically significant external validation support in the GSE119600 blood cohort. Therefore, their potential roles should be interpreted more cautiously and require further validation in independent liver tissue cohorts or experimental systems.

Overall, the external validation results suggest that CD44, FOS, and CDKN1A may represent more broadly detectable PBC-related candidate genes, whereas CCL2 may be more closely linked to the local hepatic high-risk microenvironment. This distinction is important because it indicates that the prioritized genes may not represent a single biological category, but rather different layers of PBC-related transcriptional regulation, including hepatic microenvironment-associated chemokine signaling, cell adhesion, stress activation, and cell cycle-related responses.

### Interpretation of microbial metabolite-gene associations

In recent years, the role of the gut–liver axis, particularly gut microbiota and their metabolites, in PBC has received increasing attention. Previous studies have shown that patients with PBC exhibit alterations in gut microbiota composition, and that some microbial features may be partially restored after UDCA treatment [4]. Other studies have suggested that treatment-naive patients with PBC have gut microbial profiles distinct from those of healthy individuals [5]. In addition, short-chain fatty acids and fecal microbial features have been associated with fibrosis severity in PBC, suggesting that microbial metabolites may be involved in processes related to disease progression or disease severity [6]. These findings provide a biological rationale for investigating high-risk PBC candidate targets through an integrative framework linking microbial metabolite-associated signals with host gene responses.More broadly, recent studies across chronic liver diseases have emphasized the importance of gut-derived signals and host molecular alterations in shaping hepatic disease phenotypes, supporting the application of integrative approaches that connect microbial, metabolic, and host regulatory information [21,22].

The metabolites involved in the present study included acetate, butyrate, propionate, succinate, urolithin A, trimethylamine oxide, and others. Among them, acetate, butyrate, and propionate are short-chain fatty acids that have received broad attention for their roles in immune regulation, intestinal barrier homeostasis, and host metabolism [6,7]. Succinate is often linked to inflammatory metabolic reprogramming and immune cell activation [8]. Notably, Han et al. reported that gut microbiota and metabolite signatures in patients with PBC were associated with poor biochemical response after UDCA treatment, further supporting the rationale for incorporating the “gut microbiota-metabolite-host response” axis into studies of PBC risk stratification [7]. Jiang et al. also reported, based on fecal microbiota transplantation-related experiments, that fecal microbiota from patients with PBC could induce or aggravate PBC-like liver lesions, providing more direct experimental evidence for the potential participation of gut microbiota in PBC [23].Together, these findings support the concept that integrating microbial, metabolomic, and host transcriptomic information may provide a translational framework for identifying molecular links between intestinal environmental changes and hepatic disease phenotypes in chronic liver diseases.Similar integrative molecular approaches have increasingly been applied in chronic liver disease research to explore disease mechanisms and identify potential therapeutic targets [22–24].

However, it should be emphasized that this study did not directly measure metabolite levels in serum, feces, bile, or liver tissue from patients with PBC. Therefore, the metabolite-gene associations described in this study should be understood as gutMGene-based reported associations, rather than measured metabolite-gene correlations in a PBC cohort [8]. In other words, this study addressed the question of which high-risk PBC differentially expressed genes also have reported gut microbiota-derived metabolite association evidence and PBC disease-target evidence. It did not answer which specific metabolite directly causes a specific gene alteration in PBC. This distinction is essential for avoiding overinterpretation and provides a clearer starting point for future mechanistic studies integrating direct metabolite measurements, functional validation, and multi-omics approaches.

### Strengths

The main strength of this study is the construction of a multilayer evidence integration framework for candidate target screening. Previous studies based on GSE79850 have mainly focused on transcriptomic stratification of high-risk PBC or the identification of diagnostic candidate genes. Building on this dataset, the present study further incorporated gutMGene-derived metabolite-host gene relationships and Open Targets disease-target evidence to screen candidate genes step by step. This strategy narrowed the candidate gene set to some extent and improved the biological interpretability of candidate targets [3,8,9]. The Open Targets Platform integrates multiple sources of evidence, including genetics, known drugs, expression, animal models, pathways, and systems biology, and provides a systematic knowledge framework for disease-target prioritization [9]. Using Open Targets as a source of PBC disease association evidence may help reduce the instability associated with relying only on DEG ranking.

Another strength is that this study incorporated the Human Liver Cell Atlas for cell-type localization after candidate target screening, moving the interpretation beyond a simple gene list. The pathological process of PBC involves interactions among cholangiocytes, immune cells, hepatic stellate cells, and the intrahepatic microenvironment. Therefore, identifying the cell types in which candidate genes are expressed provides important clues for understanding their potential biological context [10]. Although this study did not perform a PBC-specific single-cell reanalysis, the publicly available human liver reference atlas still provided baseline localization information for candidate gene interpretation, especially for providing reference information regarding the baseline localization tendency of CCL2 in human liver cell populations [10].

Another strength is the inclusion of an independent external validation cohort. Although GSE119600 is a whole-blood dataset and does not directly validate high-risk liver tissue expression, it allowed us to evaluate whether the prioritized genes showed general PBC-related expression differences beyond the discovery dataset. The significant upregulation of CD44, FOS, and CDKN1A in this independent cohort supports the disease relevance of part of the candidate gene set and reduces the concern that all findings were derived from a single transcriptomic dataset.

In addition, the results of this study provide a relatively clear direction for subsequent validation. Among the six core candidate genes, CCL2 showed the most convergent integrative evidence. CD44 and FOS may represent broader inflammatory or stress-related candidate nodes, whereas CDKN1A, BCL2, and MAPK3 suggest that cell cycle regulation, apoptosis regulation, and signal transduction processes may participate in the transcriptional state of high-risk PBC. Compared with simply reporting a list of DEGs, this candidate stratification is more useful for subsequent independent cohort validation, targeted qPCR or IHC validation, metabolite detection, and in vitro cell experiments.

### Limitations

Several limitations should be acknowledged. First, the sample size of GSE79850 was relatively small, and this study only compared high-risk and low-risk PBC samples without validation in an independent external liver tissue cohort [3]. In small transcriptomic studies, inter-individual variation, sample preservation, and platform-specific factors may influence some differential expression results. Therefore, the candidate genes identified in this study are more appropriate as targets for subsequent validation and should not be directly regarded as stable clinical biomarkers.

Second, high-risk PBC and inadequate UDCA response are related but should not be treated as identical concepts. The original GSE79850 study linked high-risk disease with inadequate UDCA response and risk of disease progression [3], and clinical guidelines also emphasize the importance of UDCA response assessment for identifying high-risk patients with PBC [1,2]. However, the dataset used in this study was not a large prospective cohort strictly designed according to UDCA responder and non-responder status. Therefore, the results should be interpreted in terms of high-risk PBC or high-risk status, rather than being directly described as a molecular mechanism of UDCA resistance.

Third, gutMGene provides reported microbial metabolite-host gene associations, which do not represent the actual abundance of metabolites in the samples analyzed in this study and cannot prove directional causal relationships between metabolites and gene expression in PBC [8]. Similarly, the Open Targets globalScore is an integrated disease-target evidence score and does not equal a functional causal effect or replace experimental validation [9]. The Human Liver Cell Atlas is a reference human liver single-cell atlas, not a PBC-specific single-cell cohort [10]. Therefore, the immune-cell-biased localization of CCL2 only indicates its baseline expression tendency in the human liver cellular ecosystem and cannot directly define its cellular source within PBC lesions or its disease-state expression changes. Finally, this study did not include protein-level validation, tissue localization validation, or in vitro intervention experiments, which limits further interpretation of the functional significance of the candidate targets.

These limitations do not negate the value of this study as a candidate target screening analysis, but they indicate that the conclusions should remain cautious. More precisely, this study provides a prioritized candidate target list with relatively clear validation directions, especially by identifying CCL2 as a candidate worthy of focused attention in future experimental and clinical cohort studies. It does not prove that CCL2 mediates high-risk PBC or inadequate UDCA response.

In addition, the immune signature analysis was exploratory and based on a limited number of marker genes available in the GSE79850 expression matrix. The observed chemokine signaling difference and CCL2-related correlation did not remain significant after multiple testing correction. Therefore, these results should be considered supportive evidence for the immune-inflammatory context of CCL2, rather than definitive evidence of immune cell infiltration or causal immune activation.

Although GSE119600 was used as an independent external validation dataset, it is a whole-blood transcriptomic cohort rather than a liver tissue dataset. Moreover, it compares PBC samples with controls and does not provide high-risk versus low-risk PBC stratification. Therefore, the GSE119600 analysis cannot be considered a direct validation of high-risk PBC-specific hepatic expression changes. The differences in tissue source and disease stratification between the discovery and validation cohorts should therefore be considered when interpreting both concordant and discordant validation results. The lack of consistent CCL2 upregulation in this blood dataset further indicates that CCL2 should not be interpreted as a validated peripheral blood biomarker in this study. Independent liver tissue cohorts, PBC-specific single-cell data, and spatial transcriptomic validation are still needed.

### Future directions

Future studies should first validate CCL2 in independent PBC liver tissue cohorts, because the present GSE119600 blood validation did not confirm consistent CCL2 upregulation in peripheral blood. In parallel, CD44, FOS, and CDKN1A should be further evaluated as externally supported candidates, especially in relation to UDCA response, ALP and bilirubin changes, GLOBE score, or UK-PBC score. [25,26]. Second, relevant metabolite levels in feces, serum, or bile should be measured simultaneously, including short-chain fatty acids, succinate, trimethylamine oxide, and other related metabolites, to determine whether the gutMGene-based metabolite-gene associations inferred in this study can be supported in real PBC cohorts [6,8]. Third, cholangiocytes, macrophages, or liver organoid co-culture systems could be used to examine the effects of acetate, butyrate, propionate, and succinate on CCL2 expression and downstream immune-inflammatory signals, thereby translating the database-integrated results into testable mechanistic hypotheses [8,17].

If feasible, single-cell RNA sequencing or spatial transcriptomics of PBC liver tissue would be an important direction for further validation. Single-cell or spatial data could help clarify the specific cellular source of CCL2 in PBC lesions, determine whether CCL2 is enriched around immune-infiltrated peri-biliary regions, and assess its spatial relationship with CCR2-positive monocyte/macrophage recruitment [10,17]. Overall, by integrating multiple public databases, this study proposes a high-risk PBC candidate target framework centered on gut microbiota-derived metabolite-associated host genes, with CCL2 as a representative prioritized candidate. This provides a relatively clear entry point for future mechanistic and translational studies from the perspective of the gut microbiota-metabolite-intrahepatic immune inflammation-high-risk disease state axis.

## Conclusion

This study integrated gutMGene, the GSE79850 liver transcriptomic dataset, Open Targets disease-target evidence, the Human Liver Cell Atlas, exploratory immune signature analysis, and the GSE119600 external validation cohort to construct a gut microbiota-derived metabolite-associated host target screening framework for PBC. Based on this multilayer evidence integration strategy, six core candidate genes were identified, including CCL2, CDKN1A, BCL2, CD44, FOS, and MAPK3.

Within the high-risk liver transcriptomic discovery framework, CCL2 showed the most convergent evidence. It was linked to multiple gut microbiota-related metabolites, including acetate, butyrate, propionate, and succinate, was significantly upregulated in high-risk PBC liver samples, showed PBC disease-target evidence, and displayed immune-related cell-type localization in the Human Liver Cell Atlas. Exploratory immune signature analysis further supported a chemokine-related immune-inflammatory transcriptional context for CCL2. However, CCL2 did not show consistent upregulation in the GSE119600 whole-blood validation cohort, suggesting that it may represent a hepatic microenvironment-associated candidate rather than a broadly detectable peripheral blood expression marker, particularly considering the differences in tissue source and disease stratification between the discovery and validation cohorts.

In contrast, CD44, FOS, and CDKN1A were significantly upregulated in the independent GSE119600 blood transcriptomic cohort, providing external disease-related expression support for part of the prioritized candidate gene set. These findings suggest that the candidate genes identified in this study may represent different layers of PBC-related biology, with CCL2 more closely linked to the local hepatic high-risk context and CD44, FOS, and CDKN1A showing broader cross-dataset disease relevance.

It should be emphasized that this study was an exploratory public database-based integrative screening analysis. The results cannot directly prove that gut microbiota-derived metabolites cause high-risk PBC or inadequate UDCA response, nor can they establish definitive causal mechanisms for the identified candidate genes. Future studies should combine independent liver tissue cohorts, metabolite measurements, tissue localization validation, cell experiments, and PBC-specific single-cell or spatial transcriptomic data to clarify the biological significance of these candidate targets in PBC progression and treatment response.

## Supporting information

S1 Fig Reference cell-type localization patterns of five additional candidate genes in the Human Liver Cell Atlas. UMAP visualization of the reference cell-type localization patterns of (A) CDKN1A, (B) BCL2, (C) CD44, (D) FOS, and (E) MAPK3 in the Human Liver Cell Atlas.
(TIF)

S1 Table Immune signature marker genes used in the exploratory immune signature analysis. The table lists the marker genes included in each immune-related signature used in the exploratory analysis.
(XLSX)

S1 Code R code used for the principal analyses in this study. The file contains the principal R code used for data processing, integrative analysis, visualization, immune signature analysis, and external validation.
(R)

S1 Data Processed Human Liver Cell Atlas objects used for candidate-gene localization analysis. The RData file contains the processed reference cell annotations, UMAP coordinates, cell barcodes, candidate-gene indices, and sparse candidate-gene expression records used to reproduce the HLCA-based localization summaries.
(RDATA)

## References

[1] European Association for the Study of the Liver. EASL Clinical Practice Guidelines: the diagnosis and management of patients with primary biliary cholangitis. J Hepatol. 2017;67(1):145–72. doi: 10.1016/j.jhep.2017.03.022 28427765

[2] Lindor KD, Bowlus CL, Boyer J, Levy C, Mayo M. Primary biliary cholangitis: 2018 practice guidance from the American Association for the study of liver diseases. Hepatology. 2019;69(1):394–419. doi: 10.1002/hep.30145 30070375

[3] Hardie C, Green K, Jopson L, Millar B, Innes B, Pagan S, et al. Early molecular stratification of high-risk primary biliary cholangitis. EBioMedicine. 2016;14:65–73. doi: 10.1016/j.ebiom.2016.11.021 27913155 PMC5161439

[4] Tang R, Wei Y, Li Y, Chen W, Chen H, Wang Q, et al. Gut microbial profile is altered in primary biliary cholangitis and partially restored after UDCA therapy. Gut. 2018;67(3):534–41. doi: 10.1136/gutjnl-2016-313332 28213609

[5] Zhou Y-J, Ying G-X, Dong S-L, Xiang B, Jin Q-F. Gut microbial profile of treatment-naive patients with primary biliary cholangitis. Front Immunol. 2023;14:1126117. doi: 10.3389/fimmu.2023.1126117 37223092 PMC10200865

[6] Lammert C, Shin AS, Xu H, Hemmerich C, O’Connell TM, Chalasani N. Short-chain fatty acid and fecal microbiota profiles are linked to fibrosis in primary biliary cholangitis. FEMS Microbiol Lett. 2021;368(6):fnab038. doi: 10.1093/femsle/fnab038 33836051 PMC8062329

[7] Han W, Song T, Huang Z, Liu Y, Xu B, Huang C. Distinct signatures of gut microbiota and metabolites in primary biliary cholangitis with poor biochemical response after ursodeoxycholic acid treatment. Cell Biosci. 2024;14(1):80. doi: 10.1186/s13578-024-01253-1 38879547 PMC11180406

[8] Cheng L, Qi C, Yang H, Lu M, Cai Y, Fu T, et al. gutMGene: a comprehensive database for target genes of gut microbes and microbial metabolites. Nucleic Acids Res. 2022;50(D1):D795–800. doi: 10.1093/nar/gkab786 34500458 PMC8728193

[9] Ochoa D, Hercules A, Carmona M, Suveges D, Baker J, Malangone C, et al. The next-generation Open Targets Platform: reimagined, redesigned, rebuilt. Nucleic Acids Res. 2023;51(D1):D1353–9. doi: 10.1093/nar/gkac1046 36399499 PMC9825572

[10] MacParland SA, Liu JC, Ma X-Z, Innes BT, Bartczak AM, Gage BK, et al. Single cell RNA sequencing of human liver reveals distinct intrahepatic macrophage populations. Nat Commun. 2018;9(1):4383. doi: 10.1038/s41467-018-06318-7 30348985 PMC6197289

[11] Barrett T, Wilhite SE, Ledoux P, Evangelista C, Kim IF, Tomashevsky M, et al. NCBI GEO: archive for functional genomics data sets--update. Nucleic Acids Res. 2013;41(Database issue):D991-5. doi: 10.1093/nar/gks1193 23193258 PMC3531084

[12] Davis S, Meltzer PS. GEOquery: a bridge between the Gene Expression Omnibus (GEO) and BioConductor. Bioinformatics. 2007;23(14):1846–7. doi: 10.1093/bioinformatics/btm254 17496320

[13] Ritchie ME, Phipson B, Wu D, Hu Y, Law CW, Shi W, et al. limma powers differential expression analyses for RNA-sequencing and microarray studies. Nucleic Acids Res. 2015;43(7):e47. doi: 10.1093/nar/gkv007 25605792 PMC4402510

[14] Benjamini Y, Hochberg Y. Controlling the false discovery rate: a practical and powerful approach to multiple testing. J R Stat Soc Ser B: Stat Methodol. 1995;57(1):289–300. doi: 10.1111/j.2517-6161.1995.tb02031.x

[15] Wickham H. ggplot2: Elegant graphics for data analysis. New York: Springer-Verlag; 2016. doi: 10.1007/978-3-319-24277-4

[16] Pedersen TL. ggraph: An implementation of grammar of graphics for graphs and networks. R package version 2.2.2; 2025.

[17] Reuveni D, Gore Y, Leung PSC, Lichter Y, Moshkovits I, Kaminitz A, et al. The critical role of chemokine (C-C Motif) receptor 2-positive monocytes in autoimmune cholangitis. Front Immunol. 2018;9:1852. doi: 10.3389/fimmu.2018.01852 30158929 PMC6104446

[18] Pham HN, Pham L, Sato K. Bioinformatic analysis identified novel candidate genes with the potentials for diagnostic blood testing of primary biliary cholangitis. PLoS One. 2023;18(10):e0292998. doi: 10.1371/journal.pone.0292998 37844121 PMC10578581

[19] Sutherland TE, Dyer DP, Allen JE. The extracellular matrix and the immune system: a mutually dependent relationship. Science. 2023;379(6633):eabp8964. doi: 10.1126/science.abp8964 36795835

[20] Cruz-Mendoza F, Jauregui-Huerta F, Aguilar-Delgadillo A, García-Estrada J, Luquin S. Immediate early gene c-fos in the brain: focus on glial cells. Brain Sci. 2022;12(6):687. doi: 10.3390/brainsci12060687 35741573 PMC9221432

[21] Borges-Canha M, Centelles-Lodeiro J, Leite AR, Chaves J, Lourenço IM, Von-Hafe M, et al. Gut dysbiosis is linked to severe steatosis and enhances its diagnostic performance in MASLD. eGastroenterology. 2025;3(3):e100204. doi: 10.1136/egastro-2025-100204 40917924 PMC12410686

[22] Wang S, Farokhian A, Shen B. Clinical association between inflammatory bowel disease and primary sclerosing cholangitis: what changes after colectomy and liver transplantation? eGastroenterology. 2025;3(3):e100199. doi: 10.1136/egastro-2025-100199 PMC12336565 40791845

[23] Jiang H, Yu Y, Hu X, Du B, Shao Y, Wang F, et al. The fecal microbiota of patients with primary biliary cholangitis (PBC) causes PBC-like liver lesions in mice and exacerbates liver damage in a mouse model of PBC. Gut Microbes. 2024;16(1):2383353. doi: 10.1080/19490976.2024.2383353 39105259 PMC11305030

[24] Bueloni B, Garcia Fernandez de Barrena M, Avila MA, Bayo J, Mazzolini G. Epigenetic mechanisms involved in hepatocellular carcinoma development and progression. eGastroenterology. 2025;3(2):e100186. doi: 10.1136/egastro-2025-100186 40432834 PMC12107448

[25] Lammers WJ, Hirschfield GM, Corpechot C, Nevens F, Lindor KD, Janssen HLA, et al. Development and validation of a scoring system to predict outcomes of patients with primary biliary cirrhosis receiving ursodeoxycholic acid therapy. Gastroenterology. 2015;149(7):1804-1812.e4. doi: 10.1053/j.gastro.2015.07.061 26261009

[26] Carbone M, Sharp SJ, Flack S, Paximadas D, Spiess K, Adgey C, et al. The UK-PBC risk scores: derivation and validation of a scoring system for long-term prediction of end-stage liver disease in primary biliary cholangitis. Hepatology. 2016;63(3):930–50. doi: 10.1002/hep.28017 26223498 PMC6984963

